# Polymorphisms of *KCNJ6* Gene and Their Correlation with Immune Indicators in Yaks (*Bos grunniens*)

**DOI:** 10.3390/biom14121576

**Published:** 2024-12-10

**Authors:** Wenwen Ren, Xiaoming Ma, Daoning Yu, Xiaoyun Wu, Yongfu La, Xian Guo, Min Chu, Ping Yan, Xianyong Lan, Chunnian Liang

**Affiliations:** 1 Key Laboratory of Animal Genetics and Breeding on Tibetan Plateau, Ministry of Agriculture and Rural Affairs, Lanzhou Institute of Husbandry and Pharmaceutical Sciences, Chinese Academy of Agricultural Sciences, Lanzhou 730050, Chinamaxiaoming@caas.cn (X.M.); wuxiaoyun@caas.cn (X.W.); layongfu@caas.cn (Y.L.); guoxian@caas.cn (X.G.); chumin@caas.cn (M.C.); yanping@caas.cn (P.Y.); 2 Key Laboratory of Yak Breeding Engineering of Gansu Province, Lanzhou Institute of Husbandry and Pharmaceutical Sciences, Chinese Academy of Agricultural Sciences, Lanzhou 730050, China; 3 Key Laboratory of Animal Genetics, Breeding and Reproduction of Shaanxi Province, College of Animal Science and Technology, Northwest A&F University, Xianyang 712100, China

**Keywords:** yak, *KCNJ6*, SNPs, immune indicators

## Abstract

Yaks are crucial to local herders’ economy and agriculture. However, several diseases pose a significant threat to the health of yaks and cause substantial economic losses for herders. Therefore, studying the immune indicators and breeding of yaks has become an important task. This study aimed to investigate the association between single nucleotide polymorphisms (SNPs) of the G protein-activated inwardly rectifying K+ channel 2 (*KCNJ6*, GIRK2) gene and yak immune indicators, with the goal of identifying potential candidate molecular markers for yak breeding. In this study, we genotyped 192 healthy adult yaks and detected three SNPs (g163684421 C > T, g163688148 C > T, and g163690745 T > C) in the yak *KCNJ6* gene. These SNPs were found to be distributed in the yak population. Subsequently, we performed a linkage disequilibrium analysis and found that the linkage disequilibrium levels of g163684421 C > T and g163690745 T > C were relatively high. Through a correlation analysis of yak *KCNJ6* gene SNPs and immune indicators, we found that g163684421 C > T and g163690745 T > C were significantly associated with IgA, IgG, IgM, CRP, HP, IL-2, IL-4, IFN-γ, and TNF-α (*p* < 0.05), and the mutation of these SNPs leads to a decrease in yak immune indicators. On the other hand, g163688148 C > T was significantly associated with IgG, IL-4, IFN-γ, TNF-α, IgA, CRP, and HP *(p* < 0.05), and the mutation of this SNP leads to an increase in yak immune indicators. In conclusion, we identified SNPs associated with yak immune indicators and found that *KCNJ6* gene polymorphisms can serve as candidate molecular markers for yak immune indicators. This study provides valuable genetic resources for marker-assisted selection in yak breeding. The results of this study are of great importance for the research on yak immune indicators and marker-assisted selection in yak breeding.

## 1. Introduction

G protein-activated inwardly rectifying K^+^ channels (GIRKs) belong to the KIR3 subfamily and are key regulators of neuronal excitability in the heart and neurons [1,2]. Upon activation of various G protein-coupled receptors, GIRK channels can hyperpolarize neurons, thereby modulating neuronal excitability through GIRK-mediated autoinhibition, slow synaptic potentiation, and fluid transmission. Consequently, GIRK channels may represent a valuable new therapeutic target [2]. There are four subunits of the GIRK channel in humans, designated as KIR3.1 (*KCNJ3*); KIR3.2 (*KCNJ6*, *GIRK2*); KIR3.3 (*KCNJ9*); and KIR3.4 (*KCNJ5*) [1,2]. *KCNJ6* (*GIRK2*) is a subunit of GIRK that is widely enriched in the brain and is associated with various functions and pathologies, including learning and memory, motor coordination, and Down syndrome [3]. The *KCNJ6* (*GIRK2*) gene encodes a G protein-controlled, ATP-sensitive inward-rectifying potassium channel, and the dominant mutations in the *KCNJ6* (*GIRK2*) gene have been linked to Keppen–Lubinsky syndrome, a disorder characterized by lipodystrophy, severe developmental delay, intellectual disability, hypertension, hyperreflexia, and growth retardation [4]. Research has shown that a single nucleotide substitution in the *KCNJ6* (*GIRK2*) gene can result in a change in the highly conserved Gly-Tyr-Gly sequence of the *GIRK2* protein’s pore-forming region (H5) to Ser-Tyr-Gly, leading to the manifestation of Weaver-like symptoms [5,6].

The yak (*Bos grunniens*), known as the ‘boat of the plateau’, is a native mammal of high-altitude plateaus, primarily distributed in the Qinghai–Tibet Plateau and its adjacent high-altitude, low-oxygen areas. Through prolonged natural selection, yaks have developed the ability to adapt to harsh, high-altitude environments and possess remarkable resilience. Oxidative stress can cause damage in mammalian cells, leading to cell macromolecules and organ dysfunction; reduce production performance; and even infect various diseases [7]. Meanwhile, immune stress can also affect animal behavior, metabolism, and neurosecretion, ultimately leading to growth inhibition [8]. In the traditional livestock rearing system reliant on natural grazing, yak nutrition is heavily influenced by the seasonal fluctuations in forage yield and nutrient content. Insufficient forage supply or inadequate nutrient levels can lead to prolonged nutritional deficiencies in pregnant cows and newborn calves, ultimately resulting in stunted growth and development in yaks [9]. Furthermore, the challenging environmental conditions of high-altitude grasslands contribute to a higher incidence of disease in yak calves. Calves in sub-optimal health are particularly susceptible to growth and development delays.

Immune indicators provide valuable insights into an animal’s resistance to diseases. An animal’s ability to ward off illnesses is largely determined by the health and efficacy of its immune system. While blood components typically exhibit a stable profile under normal conditions, physiological or pathological changes in the animal’s body often lead to alterations in blood cell counts and morphology. Therefore, immunological parameters derived from blood components serve as crucial indicators for assessing and evaluating an animal’s immune status and overall health [10]. Therefore, immune indicators serve as a valuable reference point for assessing the disease resistance and overall health of yaks. By closely monitoring fluctuations in these indicators, potential immune system challenges can be identified promptly. This allows for the implementation of timely interventions to enhance their resistance to diseases, ultimately preventing and managing illnesses effectively. The aim of this study is to investigate whether genetic variations in the *KCNJ6* gene contribute to the disease susceptibility of yaks in high-altitude environments and to explore the potential of immune indicators in assessing yak disease resistance and health status.

## 2. Materials and Methods

### 2.1. Ethics Approval

All the animal experiments were approved by the Lanzhou Institute of Animal Husbandry and Pharmaceutical Sciences of the Chinese Academy of Agricultural Sciences (CAAS), with grant number 1610322020018.

### 2.2. Sample Collection of Test Animals

Blood samples (5 mL each) were collected from 192 fasted 3–5-year-old Niangya female yaks at Tibet Niangya Yak Breeding Industry Development Limited Liability Company in Jiali County, Naqu City, Tibet Autonomous Region. These samples were collected into clean, clot-activation vacuum tubes and allowed to settle for 30 min before centrifugation at 3500 rpm for 10 min. The supernatant was carefully aspirated into PE tubes, sealed, and stored at −20 °C in a refrigerator.

Additionally, 5 mL of blood was collected from each yak into EDTA-K2 anticoagulant tubes. Upon completion of blood collection, the samples were thoroughly mixed, placed in a sampling box containing an ice pack, and transported promptly to a laboratory. Upon arrival, the samples were frozen at −20 °C for subsequent DNA extraction.

### 2.3. Detection of Immune Indicators

Immunoglobulin IgA (MB-4907A), IgG (MB-4616A), and IgM (MB-4908A); C-reaction protein (CRP) (MB-2161B); serum haptoglobin (HP) (MB-2158B); interleukin 2 (IL-2) (MB-4923B), interleukin 4 (IL-4) (MB-4904B), and interleukin 6 (IL-6) (MB-4905B); interferon-gamma (IFN-γ) (MB-4902B); and tumor necrosis factor-α (TNF-α) (MB-4838B) levels were measured using a double antibody one-step sandwich method, according to the detection kits of Jiangsu Enzyme Biotechnology Co., Ltd., Yancheng, China.

### 2.4. Extraction of Blood Genomic DNA

Genomic DNA was extracted from the blood samples using a blood genomic DNA extraction kit from TianGen Biochemical Technology (Beijing) Co., Ltd., Beijing, China. The extracted DNA was then assessed for concentration and purity using a UV spectrophotometer. DNA with a concentration greater than 20 ng/µL and an OD260/OD280 ratio between 1.7 and 1.9 was considered suitable for the experiment. The extracted DNA was stored at −20 °C.

### 2.5. Primer Design

To specifically amplify the regions encompassing the g163684421 C > T, g163688148 C > T, and g163690745 T > C SNP loci on chromosome 1 (ensemble accession number: ENSBGRG00000003372) of the yak genome (LU_Bosgru_v3.0 version), primers were designed using the Primer5 software. The details of these forward and reverse primers, as listed in Table 1, were synthesized by Beijing Qingke Biotechnology Co., Ltd., Beijing, China.

### 2.6. PCR Amplification and Sequencing

A 25 µL PCR amplification system was assembled using the following components: the 2 × L-Exp Taq Master Mix (dye plus), 12.5 μL; RNase free water, 8.5 μL; an upstream primer, 1 μL; a downstream primer, 1 μL; and template DNA, 2 μL. The PCR amplification program consisted of the following steps: initial denaturation at 94 °C for 1 min, followed by 35 cycles of denaturation at 98 °C for 10 s, annealing at 60 °C for 30 s, and extension at 72 °C for 1 min, with a final extension at 72 °C for 2 min. PCR products were subjected to electrophoresis on a 1% agarose gel. After confirmation of successful amplification via gel electrophoresis, the PCR products were sequenced using Sanger sequencing (JY-SPGT). Sequencing was performed by Beijing Qingsk Biotechnology Co., Ltd., Beijing, China. The bioanalysis software MEGA 11.0 was used to compare the sequencing results of PCR products and analyze the sequencing peak diagram, and the PARMS SNP genotyping technique was used to genotype the DNA of all samples.

### 2.7. Statistical Analysis

According to the genotyping results, the number of individuals with different genotypes at each locus was counted. Genotype and allele frequencies, the effective allele number (Ne), observed heterozygosity (He), polymorphism information content (PIC), and Hardy–Weinberg equilibrium were calculated using the Popgen32 software (V1.32). Associations between different genotypes and IgA, IgG, IgM, CRP, HP, IL-2, IL-4, IL-6, IFN-γ, and TNF-α levels were assessed using a general linear model analysis in the IBM SPSS Statistics 26 software (26.0.x). Results are expressed as “mean ± standard error”.

## 3. Results

### 3.1. Analysis of KCNJ6 Genotyping Results and Genetic Parameters in Yaks

**Figure 1:**
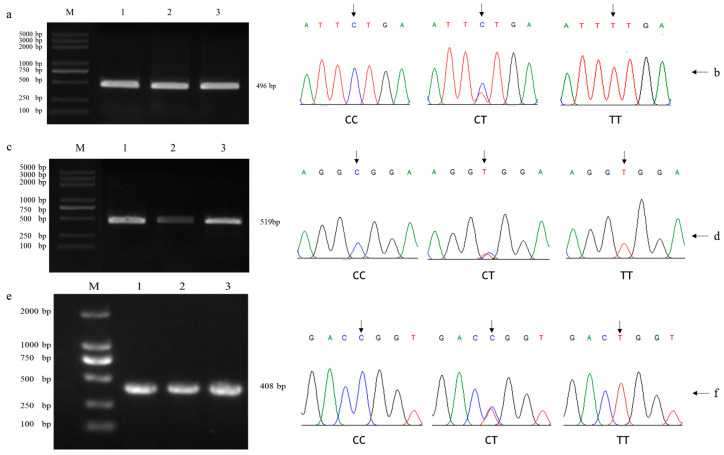
PCR amplification products and peak maps of three SNPs of *KCNJ6* gene. Note: (**a**) electrophoresis image for the g163684421 C > T SNP locus. (**b**) Fragment analysis showing the three genotypes for the g163684421 C > T SNP. (**c**) Electrophoresis image for the g163688148 C > T SNP locus. (**d**) Fragment analysis showing the three genotypes for the g163688148 C > T SNP. (**e**) Electrophoresis image for the g163690745 T > C SNP locus. (**f**) Fragment analysis showing the three genotypes for the g163690745 T > C SNP.

The amplification products of g163684421C > T, g163688148C > T, and g163690745T > C SNP loci on chromosome 1 of yaks were detected by a 1% agarose gel. Clear bands without evidence of mixed amplification were observed. The specificity of the amplification was deemed good. Fragment sizes of 496 bp, 519 bp, and 408 bp were detected, aligning with the expected sizes, thus validating the amplicons for subsequent analysis. Figure 1 displays the sequencing chromatogram and sequence data of the purified PCR product. A visual inspection of Figure 1 clearly demonstrates the presence of the T-C mutations at the g163684421C > T, g163688148C > T, and g163690745T > C SNP loci. An analysis of the chromatograms revealed three genotypes, CC, CT, and TT, for each SNP locus.

The descriptive statistics of immune traits of 192 yaks are shown in Table 2. The genotype and allele frequencies for three SNPs (g163684421C > T, g163688148C > T, and g163690745T > C) located on chromosome 1 of the yak (based on the LU_Bosgru_v3.0 genome assembly) were analyzed using population genetics principles (Table 3). All loci had calculated PIC values between 0.25 and 0.50, were moderately polymorphic in the population analyzed, and had an MAF above 5%. The genotype distribution of all loci was in Hardy–Weinberg equilibrium.

### 3.2. Linkage Disequilibrium Analysis of KCNJ6 Gene SNP in Yaks

**Figure 2:**
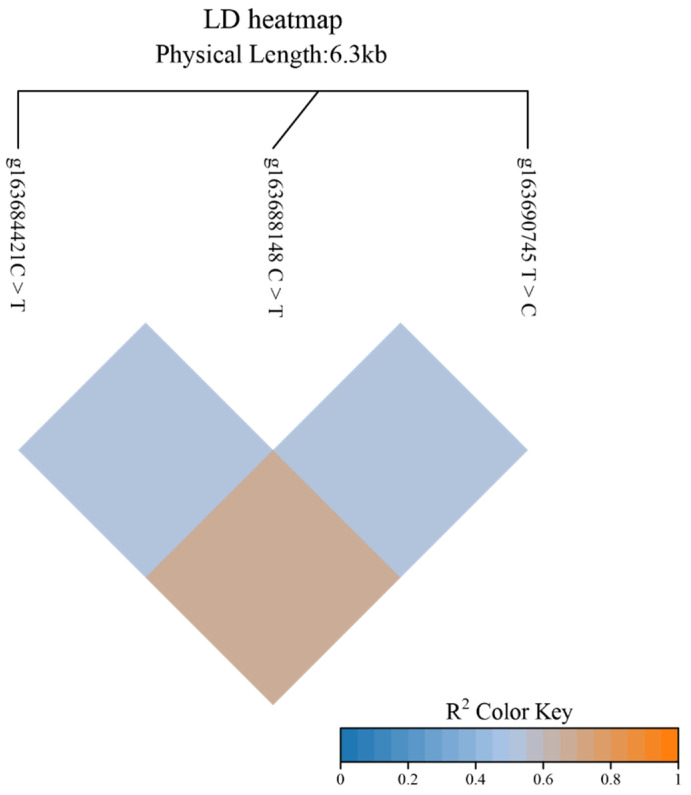
Linkage disequilibrium analysis among three SNPs of *KCNJ6* gene.

A linkage disequilibrium (LD) analysis of the g163684421 C > T, g163688148 C > T, and g163690745 T > C loci in the yak *KCNJ6* gene was conducted using online tools (https://www.bioinformatics.com.cn/ (the last visit date was 17 October 2024)). The results show that g163684421 C > T and g163688148 C > T, g163684421 C > T and g163690745 T > C, and g163688148 C > T and g163690745 T > C had linkage disequilibrium. Furthermore, a relatively strong degree of LD was observed between g163684421 C > T and g163690745 T > C (Figure 2).

### 3.3. Association Analysis Between Immune Indicators and SNP Genotypes in Yaks

Correlations between different yak genotypes and immunoglobulin levels (IgA, IgG, IgM, CRP, HP, IL-2, IL-4, IL-6, IFN-γ, and TNF-α) were investigated using a general linear model in the IBM SPSS Statistics 26 software. The results show that the g163684421 C > T SNP locus was significantly associated with IgA, IgG, IgM, CRP, HP, IL-4, IFN-γ, TNF-α in yaks (*p* < 0.01) and was significantly associated with IL-2 (*p* < 0.05). Specifically, the CC genotype demonstrated significantly higher expression levels compared to the TT genotype for these immune markers. A significant association between the g163688148 C > T SNP locus and IgG, IL-4, IFN-γ, and TNF-α was observed (*p* < 0.01). Additionally, IgA, CRP, and HP showed significant associations with this locus (*p* < 0.05). Notably, the TT genotype exhibited significantly higher expression levels relative to the CC genotype for these immune markers. Significant associations existed between the g163690745 T > C SNP locus and IgA, CRP, HP, IL-4, IFN-γ, and TNF-α (*p* < 0.01). IgG, IgM, and IL-2 also exhibited significant associations with this locus (*p* < 0.05). The TT genotype demonstrated significantly higher expression levels compared to the CC genotype. These findings suggest that the three SNPs within the yak *KCNJ6* gene play roles in regulating immune response. The nature of the association (increased or decreased immune index) differed depending on the specific SNP and genotype. (Table 4).

## 4. Discussion

### 4.1. Genetic Polymorphism Analysis of KCNJ6 Gene in Yaks

Genes and the environment interact to shape an organism’s phenotypic traits, and genetic diversity underpins the fundamental basis of immune function. Understanding immune-related factors is crucial for investigating immune responses and pathological changes in cattle during infectious and non-infectious diseases. Single nucleotide polymorphisms (SNPs) represent a primary type of candidate gene polymorphism, and SNP analysis has emerged as a powerful tool for identifying associations between candidate genes and economically important traits in livestock populations [11]. A higher PIC value indicates a greater number of alleles and heterozygous genotypes, signifying higher genetic variation and selection potential within the experimental population [12,13]. An analysis of the polymorphic information content (PIC) of the three loci revealed moderate polymorphism (0.25 < PIC < 0.5) for g163684421 C > T, g163688148 C > T, and g163690745 T > C. This suggests a high degree of variation and selection potential at these loci within the yak population. Furthermore, all three loci were found to be in Hardy–Weinberg equilibrium, indicating that these SNPs within the *KCNJ6* (*GIRK2*) gene possess certain genetic advantages. They appear to be relatively unaffected by mutations, selective pressures, and genetic drift during long-term evolution and natural selection [14]. It is noteworthy that all three SNPs are located within intronic regions of the *KCNJ6* (*GIRK2*) gene. Although intronic SNPs do not directly alter the amino acid sequence of a protein, they may indirectly influence biological processes like splicing sites, mRNA stability, or translation efficiency, potentially leading to changes in amino acid coding [15,16,17,18]. Intronic mutations have been shown to affect economically important traits in livestock, such as growth characteristics [19], fat accumulation [20], and feed efficiency [21]. Therefore, these SNPs can be considered potential candidate loci for genetic improvement in yaks.

### 4.2. Correlation Analysis of KCNJ6 Gene Polymorphisms and Immune Indicators in Yaks

An animal’s overall defense capabilities against diseases can be represented by general immune resistance. Cytokine levels indicate the body’s immune response status, indirectly reflecting the animal’s resistance to diseases [22]. Therefore, measuring cytokine levels in serum can offer an indirect reflection of the animal’s general immune resistance. Several crucial cytokines, such as interleukin and tumor necrosis factor, can assess an animal’s general immune resistance. Immunoglobulins (Ig) are essential components of the body’s defense against diseases. They are secreted by B lymphocytes in jawed vertebrates during humoral immunity, enabling the effective identification and elimination of antigens [23]. Mammals possess five Ig heavy-chain genes (μ, δ, γ, ε, and α) and two light-chain genes (κ and λ). The complete Ig molecule is formed by a combination of different heavy and light chains, designated as IgM, IgG, IgA, IgD, and IgE, respectively [24]. Helper T cells (Th) are categorized into Th1 and Th2 subsets. Th1 cells contribute to cell-mediated immunity, primarily producing IL-2 and IFN-γ. IFN-γ stimulates macrophages to generate large amounts of nitric oxide, directly killing pathogens [25]. TNF-α mediates the activation of NF-κB, which acts as a rapidly responding transcription factor, regulating the activation and expression of genes encoding pro-inflammatory cytokines, chemokines, and adhesion molecules [26,27,28]. C-reactive protein (CRP) is a quintessential acute-phase reactant with a cyclic pentameric structure. It is an ancient and highly conserved member of the pentraxin family. CRP directly participates in various inflammatory processes and contributes to the innate host immune response [29]. Furthermore, CRP serves as a sensitive marker of systemic inflammation and tissue damage, exhibiting elevated levels in patients with infections, inflammatory diseases, or necrosis [30]. Studies indicate that during the acute phase of inflammation, serum concentrations of acute-phase reactant proteins can rapidly increase from 2 to 3 times to hundreds of times [31]. Haptoglobin (HP), another acute-phase reactant, plays a vital role in the body’s response to infection, tissue damage repair, and maintaining internal homeostasis [32]. HP expression is primarily regulated by interleukin-6 (IL-6). When cows experience ketoacidosis, which exerts stress on the body, endotoxins stimulate white blood cells to produce interleukin-1 (IL-1) and interleukin-6 (IL-6). Hepatocytes express abundant haptoglobin receptors, and IL-6 promotes haptoglobin synthesis [33]. Research has shown that interleukin-4 (IL-4) exhibits immunomodulatory effects in various immune cells, including macrophages, B cells, and T cells. Its role is particularly significant in allergic inflammation [34]. IL-2 promotes the proliferation and differentiation of T and B lymphocytes and NK cells, facilitating the secretion of interferon (IFN) and tumor necrosis factor (TNF) cytokines. This boosts both cellular and humoral immune responses in animals [35]. In this study, we report that three polymorphic loci within the *KCNJ6* (*GIRK2*) gene contribute to variations in immune parameters in yaks, namely, g163684421 C > T, g163688148 C > T, and g163690745 T > C. Moreover, g163684421 C > T and g163690745 T > C mutations resulted in reduced immune parameters in yaks, suggesting that these mutations may contribute to a decreased immune capacity. Conversely, the g163688148 C > T mutation led to increased immune parameters, indicating a potential enhancement in immune function. At present, most studies have shown that variations in the *KCNJ6* gene are associated with persistent breast pain after breast cancer surgery, post-operative analgesia, and pain sensitivity [36,37]. But there are no reports on other species. Collectively, these findings highlight that the three SNPs within the *KCNJ6* gene exert diverse and significant effects on immune parameters in yaks.

## 5. Conclusions

This study preliminarily investigated the impact of three SNPs in the *KCNJ6* gene of yaks on immune indicators. The results demonstrate a significant correlation between these three SNPs and yak immune parameters. Specifically, the g163684421 C > T and g163690745 T > C mutations were associated with decreased immune indices in yaks, while the g163688148 C > T mutation led to increased immune indices. These findings suggest that the *KCNJ6* gene may serve as a potential candidate gene influencing immune parameters in yaks. This study lays a theoretical foundation for yak disease prevention and treatment, as well as the breeding of yak breeds with enhanced disease resistance.

## Figures and Tables

**Table 1:** Primer information.

SNPs	Primer Pair Sequences 1 (5′-3′)	Amplicon Length (bp)	Tm (°C)
g163684421 C > T	F: GCAGCAGGTCCGTCCACAT	496	61.2
	R: GTAGCCCATCCAGGCCACA		
g163688148 C > T	F: ACTAAGATCCCATGAGCCA	519	52.4
	R: ATTGAATGAGACACCACCAG		
g163690745 T > C	F: GCGACCTCCTCATTCTCCT	408	54.3
	R: TGTACCTTCTTCCACCCAC		

**Table 2:** Descriptive statistics of yak immune traits.

Trait	Mean	Standard Error	Maximum	Minimum
IgA (μg/mL)	1155.22	13.98	2163.35	538.09
IgG (mg/mL)	2.38	0.05	5.13	0.19
IgM (μg/mL)	2112.42	20.91	3247.17	929.93
CRP (mg/L)	13.50	0.16	21.04	3.67
HP (ng/mL)	399.70	4.37	641.62	167.36
IL-2 (pg/mL)	310.37	4.06	542.28	132.27
IL-4 (pg/mL)	67.32	1.11	100.10	13.27
IL-6 (pg/mL)	433.27	5.23	706.11	162.48
IFN-γ (pg/mL)	1742.22	18.37	2664.85	567.67
TNF-α (pg/mL)	80.73	0.90	117.10	29.43

**Table 3:** Three SNP markers of yak *KCNJ6* gene and description of related population parameters.

SNPs	Position	Genotypic Frequencies			Allelic Frequencies		χ^2^	p	PIC	He	Ne
		CC	CT	TT	C	T					
g163684421 C > T	Intron	0.239	0.426	0.335	0.452	0.548	3.741	0.053	0.373	0.495	1.982
g163688148 C > T	Intron	0.448	0.422	0.130	0.659	0.341	0.727	0.394	0.348	0.449	1.816
g163690745 T > C	Intron	0.311	0.442	0.247	0.532	0.468	2.394	0.122	0.374	0.498	1.992

**Table 4:** Correlation analysis between three SNPs of *KCNJ6* gene and immune traits of yaks.

Trait	Genotype			*p*-Value
	CC	CT	TT	
g163684421 C > T				
IgA (μg/mL)	1232.96 ± 34.57 ^a^	1155.69 ± 18.71 ^b^	1096.92 ± 22.46 ^b^	0.001 **
IgG (mg/mL)	2.67 ± 0.10 ^a^	2.29 ± 0.07 ^b^	2.26 ± 0.08 ^b^	0.002 **
IgM (μg/mL)	2207.93 ± 42.35 ^a^	2112.79 ± 28.10 ^ab^	2041.07 ± 40.25 ^b^	0.013 **
CRP (mg/L)	14.32 ± 0.32 ^a^	13.60 ± 0.20 ^a^	12.76 ± 0.29 ^b^	0.001 **
HP (ng/mL)	426.39 ± 9.26 ^a^	399.08 ± 5.37 ^b^	379.95 ± 8.62 ^b^	<0.001 **
IL-2 (pg/mL)	320.35 ± 10.18 ^a^	315.57 ± 5.52 ^a^	294.49 ± 6.24 ^b^	0.025 *
IL-4 (pg/mL)	70.51 ± 2.32 ^a^	70.47 ± 1.46 ^a^	61.25 ± 2.06 ^b^	<0.001 **
IL-6 (pg/mL)	446.14 ± 12.58	434.21 ± 7.31	421.68 ± 8.87	0.218
IFN-γ (pg/mL)	1894.67 ± 38.02 ^a^	1732.04 ± 21.35 ^b^	1648.00 ± 35.62 ^b^	<0.001 **
TNF-α (pg/mL)	83.28 ± 1.83 ^a^	82.94 ± 1.22 ^a^	75.98 ± 1.66 ^b^	0.001 **
g163688148 C > T				
IgA (μg/mL)	1119.21 ± 19.01 ^b^	1169.21 ± 22.05 ^ab^	1233.76 ± 42.62 ^a^	0.023 *
IgG (mg/mL)	2.28 ± 0.07 ^b^	2.35 ± 0.06 ^b^	2.78 ± 0.16 ^a^	0.003 **
IgM (μg/mL)	2069.59 ± 32.82	2129.82 ± 30.65	2203.38 ± 52.87	0.098
CRP(mg/L)	13.05 ± 0.22 ^b^	13.70 ± 0.24 ^ab^	14.38 ± 0.43 ^a^	0.013 *
HP (ng/mL)	389.72 ± 6.51 ^b^	401.84 ± 6.86 ^b^	426.72 ± 9.89 ^a^	0.024 *
IL-2 (pg/mL)	300.68 ± 4.98	320.81 ± 6.86	309.85 ± 13.04	0.069
IL-4 (pg/mL)	63.57 ± 1.61 ^b^	70.47 ± 1.65 ^a^	69.99 ± 3.25 ^a^	0.009 **
IL-6 (pg/mL)	429.15 ± 7.32	432.87 ± 8.39	448.56 ± 15.63	0.500
IFN-γ (pg/mL)	1671.79 ± 27.44 ^b^	1763.28 ± 26.55 ^b^	1916.24 ± 42.43 ^a^	<0.001 **
TNF-α (pg/mL)	77.71 ± 1.33 ^b^	83.42 ± 1.34 ^ab^	82.42 ± 2.44 ^a^	0.009 **
g163690745 T > C				
IgA (μg/mL)	1225.55 ± 32.19 ^a^	1153.40 ± 20.09 ^b^	1100.64 ± 22.19 ^b^	0.004 **
IgG (mg/mL)	2.61 ± 0.10 ^a^	2.30 ± 0.07 ^b^	2.30 ± 0.08 ^b^	0.018 *
IgM (μg/mL)	2197.82 ± 39.71 ^a^	2103.37 ± 30.74 ^ab^	2051.51 ± 39.65 ^b^	0.034 *
CRP (mg/L)	14.36 ± 0.30 ^a^	13.49 ± 0.23 ^b^	12.82 ± 0.28 ^b^	0.001 **
HP (ng/mL)	423.74 ± 7.70 ^a^	396.82 ± 6.47 ^b^	385.48 ± 8.36 ^b^	0.004 **
IL-2 (pg/mL)	324.88 ± 10.04 ^a^	312.49 ± 5.88 ^ab^	295.09 ± 5.96 ^b^	0.023 *
IL-4 (pg/mL)	69.93 ± 2.15 ^a^	69.40 ± 1.67 ^a^	61.74 ± 1.94 ^b^	0.004 **
IL-6 (pg/mL)	449.09 ± 11.40	430.54 ± 7.76	425.15 ± 9.05	0.215
IFN-γ(pg/mL)	1890.97 ± 33.34 ^a^	1721.55 ± 24.24 ^b^	1658.28 ± 35.59 ^b^	<0.001 **
TNF-α(pg/mL)	84.49 ± 1.68 ^a^	81.65 ± 1.34 ^a^	76.20 ± 1.58 ^b^	0.002 **

Note: ^a,b^ denote significance at *p* < 0.05. * denotes significance at *p* < 0.05, ** denotes significance at *p* < 0.01.

## Data Availability

The original contributions presented in the study are included in the article, and further inquiries can be directed to the corresponding author.
